# Synthesis of Peptidyl-tRNA Mimics for Structural Biology Applications

**DOI:** 10.1021/acs.accounts.3c00412

**Published:** 2023-09-20

**Authors:** Yury S. Polikanov, Mélanie Etheve-Quelquejeu, Ronald Micura

**Affiliations:** § Department of Biological Sciences, University of Illinois at Chicago, Chicago, Illinois 60607, United States; $ Department of Pharmaceutical Sciences, University of Illinois at Chicago, Chicago, Illinois 60607, United States; + Center for Biomolecular Sciences, University of Illinois at Chicago, Chicago, Illinois 60607, United States; ⊥ Université Paris Cité, CNRS, Laboratoire de Chimie et Biochimie Pharmacologiques et Toxicologiques, Paris F-75006, France; † Institute of Organic Chemistry and Center for Molecular Biosciences, University of Innsbruck, Innrain 80-82, 6020 Innsbruck, Austria

## Abstract

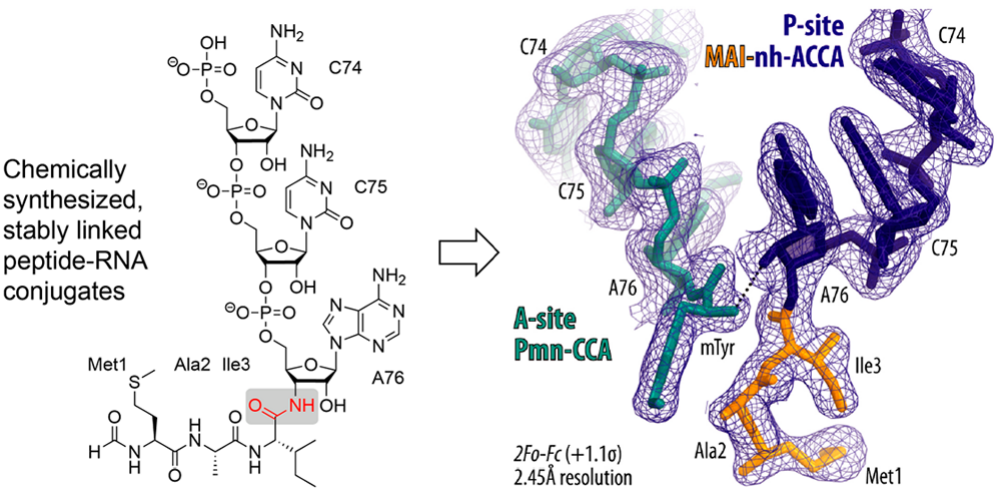

Protein biosynthesis is a central process in all living cells that is catalyzed by a complex molecular machine—the ribosome. This process is termed translation because the language of nucleotides in mRNAs is translated into the language of amino acids in proteins. Transfer RNA (tRNA) molecules charged with amino acids serve as adaptors and recognize codons of mRNA in the decoding center while simultaneously the individual amino acids are assembled into a peptide chain in the peptidyl transferase center (PTC). As the nascent peptide emerges from the ribosome, it is threaded through a long tunnel referred to as a nascent peptide exit tunnel (NPET). The PTC and NPET are the sites targeted by many antibiotics and are thus of tremendous importance from a biomedical perspective and for drug development in the pharmaceutical industry.

Researchers have achieved much progress in characterizing ribosomal translation at the molecular level; an impressive number of high-resolution structures of different functional and inhibited states of the ribosome are now available. These structures have significantly contributed to our understanding of how the ribosome interacts with its key substrates, namely, mRNA, tRNAs, and translation factors. In contrast, much less is known about the mechanisms of how small molecules, especially antibiotics, affect ribosomal protein synthesis. This mainly concerns the structural basis of small molecule–NPET interference with cotranslational protein folding and the regulation of protein synthesis. Growing biochemical evidence suggests that NPET plays an active role in the regulation of protein synthesis.

Much-needed progress in this field is hampered by the fact that during the preparation of ribosome complexes for structural studies (i.e., X-ray crystallography, cryoelectron microscopy, and NMR spectroscopy) the aminoacyl- or peptidyl-tRNAs are unstable and become hydrolyzed. A solution to this problem is the application of hydrolysis-resistant mimics of aminoacyl- or peptidyl-tRNAs.

In this Account, we present an overview of synthetic methods for the generation of peptidyl-tRNA analogs. Modular approaches have been developed that combine (*i*) RNA and peptide solid-phase synthesis on 3′-aminoacylamino-adenosine resins, (*ii*) native chemical ligations and Staudinger ligations, (*iii*) tailoring of tRNAs by the selective cleavage of natural native tRNAs with DNAzymes followed by reassembly with enzymatic ligation to synthetic peptidyl-RNA fragments, and (*iv*) enzymatic tailing and cysteine charging of the tRNA to obtain modified CCA termini of a tRNA that are chemically ligated to the peptide moiety of interest. With this arsenal of tools, in principle, any desired sequence of a stably linked peptidyl-tRNA mimic is accessible. To underline the significance of the synthetic conjugates, we briefly point to the most critical applications that have shed new light on the molecular mechanisms underlying the context-specific activity of ribosome-targeting antibiotics, ribosome-dependent incorporation of multiple consecutive proline residues, the incorporation of d-amino acids, and tRNA mischarging.

Furthermore, we discuss new types of stably charged tRNA analogs, relying on triazole- and squarate (instead of amide)-linked conjugates. Those have pushed forward our mechanistic understanding of nonribosomal peptide synthesis, where aminoacyl-tRNA-dependent enzymes are critically involved in various cellular processes in primary and secondary metabolism and in bacterial cell wall synthesis.

## Key References

- MoroderH.; StegerJ.; GraberD.; FausterK.; TrapplK.; MarquezV.; PolacekN.; WilsonD. N.; MicuraR.Non-hydrolyzable RNA-peptide conjugates: a powerful advance in the synthesis of mimics for 3′-peptidyl tRNA termini. Angew. Chem., Int. Ed.2009, 48, 4056–406010.1002/anie.20090093919396850.^1^*General concept for the solid-phase synthesis of stable peptidyl-tRNA analogs*.
- GeiermannA. S.; PolacekN.; MicuraR.Native chemical ligation of hydrolysis-resistant 3′-peptidyl-tRNA mimics. J. Am. Chem. Soc.2011, 133, 19068–1907110.1021/ja209053b22050598.^2^*First concept for chemical ligations of peptidyl-tRNA analogs*.
- GraberD.; MoroderH.; StegerJ.; TrapplK.; PolacekN.; MicuraR.Reliable semi-synthesis of hydrolysis-resistant 3′-peptidyl-tRNA conjugates containing genuine tRNA modifications. Nucleic Acids Res.2010, 38, 6796–80210.1093/nar/gkq50820525967PMC2965236.^3^*First approach for stable peptidyl-full-length tRNA analogs; usage of DNA enzymes to tailor wild-type tRNAs*.
- SyroeginE. A.; FlemmichL.; KlepackiD.; Vazquez-LaslopN.; MicuraR.; PolikanovY. S.Structural basis for the context-specific action of the classic peptidyl transferase inhibitor chloramphenicol. Nat. Struct. Mol. Biol.2022, 29, 152–16110.1038/s41594-022-00720-y35165455PMC9071271.^4^*Key reference for the application of peptidyl-tRNA analogs in ribosome structure determination*.

## 1 Introduction

**Figure 1:**
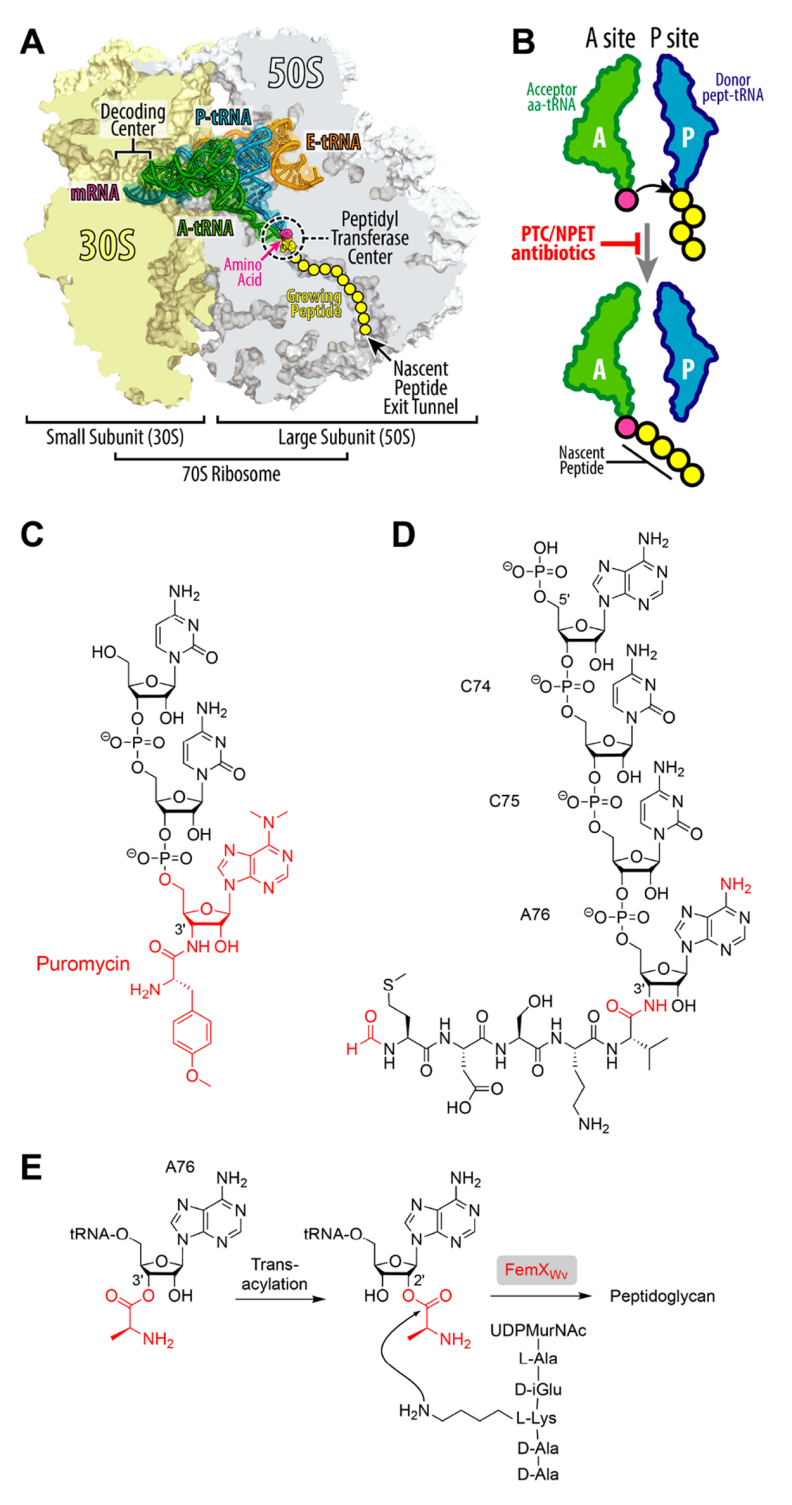
Stable peptidyl-tRNA analogs are key compounds in studies on ribosomal translation. (**A**) Functional elements of the bacterial ribosome. The growing polypeptide chain in the nascent polypeptide exit tunnel (NPET) is schematically depicted with yellow circles. (**B**) Various ribosome-targeting antibiotics inhibit the peptide bond-formation reaction catalyzed by the peptidyl transferase center (PTC). (**C**) First generation of stable small molecule 3′-peptidyl tRNA analogs was based on the antibiotic puromycin (highlighted in red). (**D**) Second generation of 3′-peptidyl-tRNA analogs that is not restricted to a tyrosine side chain for the peptide C-terminus and provides nonmethylated A76. Both generations comprise a hydrolysis-resistant 3′-amide linkage instead of the native ester. (**E**) Aminoacyl-tRNAs are also involved in nonribosomal natural product biosynthesis relying on aminoacyl-tRNA-dependent enzymes. An example reaction catalyzed by the Fem enzyme family of peptidoglycan synthesis in bacteria is shown (FemX_Wv_).

Protein biosynthesis, also known as translation, is a complex multistep process essential to every cell and is catalyzed by the ribosomes, which are molecular machines responsible for translating mRNA into proteins.^5−7^ Within the ribosome, tRNAs sequentially decode the mRNA in the decoding center of the small ribosomal subunit, while the corresponding amino acids assemble into a peptide chain in the peptidyl transferase center (PTC) in the order specified by the nucleotide sequences of the translated mRNAs (Figure 1A). Newly made proteins emerge from the ribosome through the 100-Å-long nascent peptide exit tunnel (NPET) that spans the body of the large ribosomal subunit. Initially, the NPET was thought to serve as a passive conduit for growing polypeptide chains. However, recent evidence suggests that the NPET plays an active role in cotranslational protein folding and the regulation of protein synthesis.^8,9^

PTC and NPET are sites targeted by many antibiotics. For instance, macrolides bind in the NPET of bacterial ribosomes, blocking the progression of specific growing polypeptides and causing translation to stall (Figure 1B).^10−12^ Additionally, various small organic compounds interact with the tunnel, influencing the translation rate.^8,13^ Typically, these effects are associated with specific nascent peptide sequences. Some ribosome complexes with arrested nascent peptides, mRNA, tRNAs, and antibiotics have been structurally characterized, providing insights into the conformational arrangements and interactions responsible for the tunnel’s impact on translation.^14−19^ Furthermore, the synthesis of certain peptide sequences by ribosomes is inherently challenging, as exemplified by consecutive prolines. The restricted conformational flexibility of polyprolines leads to steric hindrances, contributing to the difficulties associated with their synthesis.^20^ Overall, the emerging understanding of the ribosomal tunnel’s active role in protein synthesis and folding, as well as its modulation by various compounds, sheds light on the intricate mechanisms underlying this fundamental cellular process.

Previous structural and biochemical studies utilized the *cis*-generated peptidyl-tRNAs in the stalled ribosomal complexes to investigate the ribosome’s peptidyl transferase activity and its ability to stall in the presence of various drugs or small molecules within the NPET.^14−17^ This approach yielded ribosome nascent chain complexes (RNCs) carrying native (wild-type) peptide chains with peptidyl-tRNAs at the P site. While offering certain advantages, such as the ability to generate stable complexes, this method allows the preparation of only the peptide-arrested RNCs, representing an inactive state of the PTC by definition and not allowing for variations in the peptide sequence. Such variations are essential for comparative purposes because the inability to perform pairwise comparisons of structures of arrested versus nonarrested RNCs (e.g., with versus without antibiotic or a small molecule; WT stalling motifs versus mutants) hampers our profound understanding of the underlying ribosome stalling mechanisms.

Recently, significant progress has been made in determining the first structures of nonarrested nascent peptides in the prepeptidyl transfer state using hydrolysis-resistant full-length aminoacyl- and peptidyl-tRNAs^21^ or their short analogs.^4,21^ These analogs have been known for a long time and essentially are conjugates between a short ACCA-oligoribonucleotide and a peptide moiety. In the simplest case, such conjugates can be derived from the antibiotic puromycin (Figure 1C). The breakthrough was made possible by developing efficient procedures for preparing amide-linked peptidyl-tRNA mimics to prevent their spontaneous deacylation during experiments. Importantly, nonhydrolyzable peptidyl-tRNAs (Figure 1D) are structurally indistinguishable from native full-length tRNA substrates and retain activity in the transpeptidation reaction when combined with native aminoacyl-tRNA in the P site.^22−24^

Furthermore, it has been demonstrated that synthetic peptidyl-tRNAs and their short mimics can be effectively complexed with the ribosome *in vitro*, representing a functionally significant state of the PTC.^4,20^ Therefore, using amide-linked peptidyl-tRNA mimics reasonably approximates the reactive state, providing a reliable foundation for proposing mechanistic hypotheses. These advances open new avenues for investigating the dynamic processes and mechanisms underlying protein synthesis within the ribosome.

Short peptidyl-tRNA mimics can also be used to explore nonribosomal peptide synthesis. Aminoacyl-tRNA(aa-tRNA)-dependent enzymes are involved in various cellular processes of primary and secondary metabolism.^25^ For example, the Fem transferases are crucial for the synthesis of the bacterial cell wall; they catalyze the formation of amide and peptide bonds during peptidoglycan biosynthesis (Figure 1E).^26^ Mechanistically, the reaction proceeds through a tetrahedral intermediate that can be adequately mimicked by stably linked peptidyl-tRNA analogs. Moreover, these analogs potentially could be used as enzyme inhibitors and therefore are extremely valuable for enzyme structure determination, inhibitor design, and optimization.

## 2 Chemical Solid-Phase Synthesis of 3′-Peptidyl-tRNA Mimics

### 2.1 Basic Concept Building on 3′-Aminoacylamino-3′-deoxyadenosine Resins

The first small-molecule analogs of peptidyl-tRNAs described in the literature were based on puromycin (Pmn) as the connecting module between the peptide and the RNA moieties.^27^ For instance, CC-Pmn, ACC-Pmn, and derivatives thereof were synthesized to mimic the C74-C75-A76-amino acid terminus of a tRNA charged with a single amino acid (Figure 1C).^27,28^ Since these conjugates possess a ribose 3′-amide linkage that is relatively stable toward hydrolysis compared to the naturally occurring ester linkage,^29^ they could be successfully cocrystallized with the ribosomes and their X-ray structures could be determined at high resolution, providing important insights into the interactions between tRNA functional termini and the PTC.^30^ Moreover, peptide bond formation transition-state analogs were synthesized based on the Pmn building block,^30−34^ and the binding of corresponding derivatives to the PTC was successfully visualized. These structures provided the first insights into the mechanism of peptide bond formation catalyzed by the PTC.^30,35−37^ However, the use of a puromycin scaffold for synthesizing peptidyl-tRNA conjugates has limitations. It consists of an *O*-methylated tyrosine linked to an *N*^6^,*N*^6^-dimethyl-3′-amino-3′-deoxyadenosine moiety, both of which are not native, with *N*^6^-dimethylation at A76 and *O*-methylation at the tyrosine side chain (Figure 1C). Even more troublesome is the presence of the modified tyrosine, which restricts the C-terminus of any target peptide sequence to this particular side chain.

**Figure 2:**
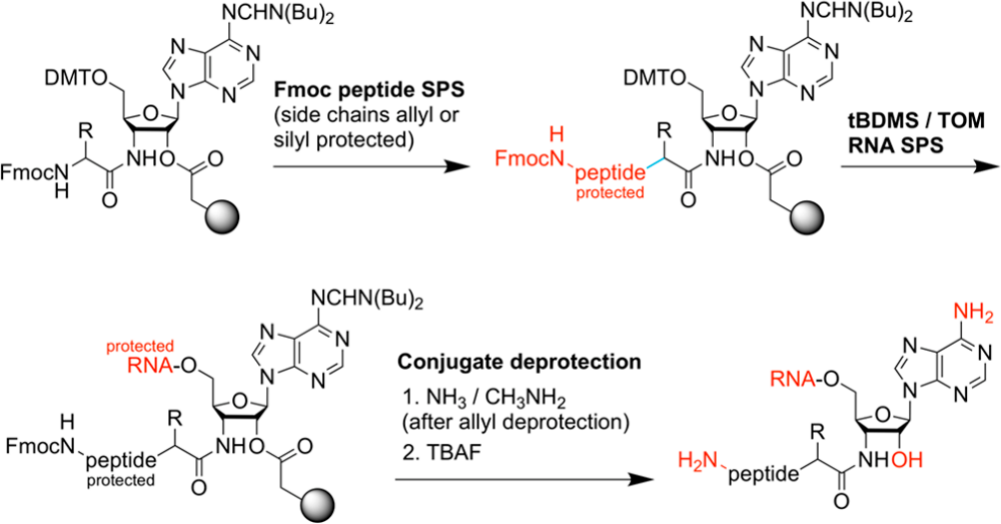
Solid-phase synthesis (SPS) of peptidyl-tRNA mimics with amide linkages based on 3′-aminoacylamino-3′-deoxyadenosine resins.^1,38^

Therefore, approaches toward the synthesis of peptidyl-tRNA conjugates that implement adenine instead of *N*^6^-dimethylated adenine and do not limit the C-terminus of the peptide chain to *O*-methylated tyrosine came into focus early on (Figure 1D). In 2003, Strazewski and co-workers introduced a concept that makes use of appropriately protected 3′-alanylamino-3′-deoxyadenosine immobilized on polystyrene resin,^38^ followed by coupling of Fmoc-protected amino acids to build up the peptide moiety (Figure 2). Subsequently, coupling of 2′-*O*-silyl-protected nucleoside phosphoramidites generates the RNA chain, and finally, the whole conjugate becomes deprotected and cleaved from the solid support. This approach follows standard peptide and standard RNA solid-phase protocols with one limitation: the peptide side chains cannot be protected with acid-labile groups typically used for Fmoc amino acid building blocks (e.g., *tert*-butyl (Ser, Thr), trityl (Cys)) and must be protected with orthogonal groups (e.g., allyl (Asp, Glu), allyloxycarbonyl (Lys, His)) or silyl groups (Ser, Thr)).

**Figure 3:**
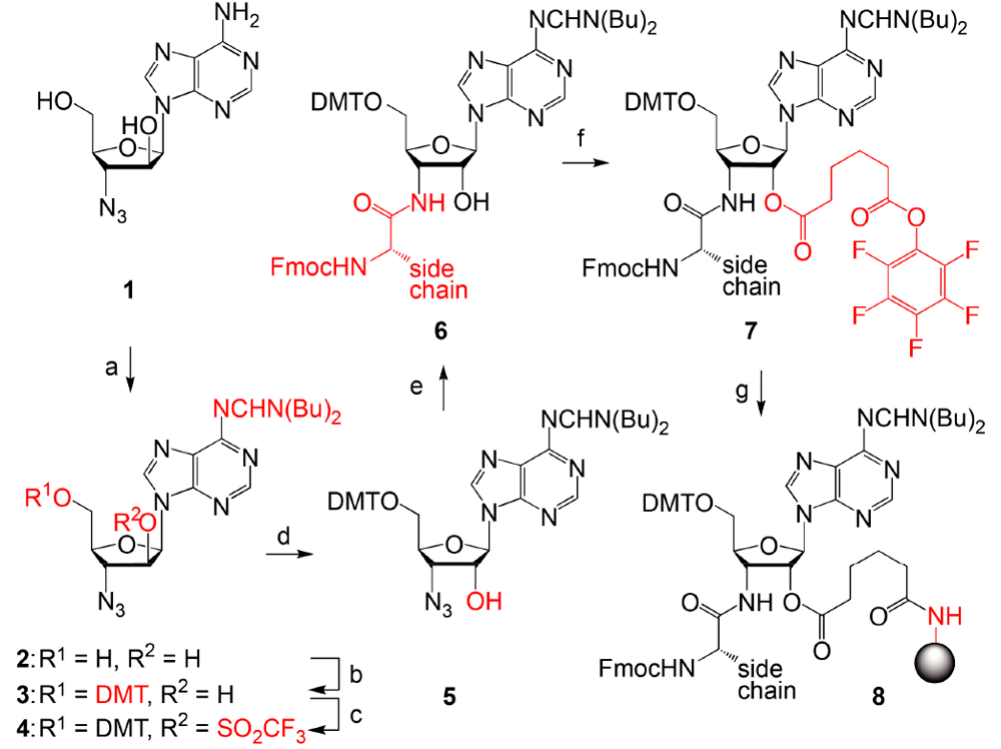
Synthesis of 3′-amino-3′-deoxyadenosine-functionalized support **8** (rA^3′-NH^) for the automated solid-phase synthesis of RNA-peptide conjugates.^1^ Reagents and conditions: (a) 4 equiv of *N*,*N*-di-*n*-butylformamide dimethyl acetal, in DMF, 80 °C, 2.5 h, 89%; (b) 2 equiv of DMT-Cl, in pyridine, room temperature,1.5 h, 79%; (c) 1.5 equiv of trifluoromethanesulfonyl chloride, 1.5 equiv of DMAP, 2.5 equiv of (*i*Pr)_2_NEt, in CH_2_Cl_2_, 0 °C, then 30 °C, 15 min; (d) 5.5 equiv of CF_3_COO^–^K^+^, 1.5 equiv of (*i*Pr)_2_NEt, 2 equiv of 18-crown-6, in toluene, 80 °C, 2.5 h (68% over (c) and (d)); (e) 1.3 equiv of Fmoc-*AA*-OBt, 2.2 equiv of P(CH_3_)_3_, in THF, 0° to room temperature, 16 h, 89%; (f) 5 equiv of PfpOOC(CH_2_)_4_COOPfp, 1 equiv of DMAP, in DMF/pyridine = 1/1, room temperature, 1 h, 71%; (g) 3 equiv of (w/w) amino-functionalized support (e.g., GE Healthcare, Custom Primer Support 200 Amino), 2 equiv of pyridine, DMF, room temperature, 22 h, loading: 40–50 μmol/g.

We (R.M. and co-workers) put substantial effort into developing the solid-phase approach for peptidyl-RNA conjugates further toward broad applicability. First, we improved synthetic access to aminoacylamino-3′-deoxyadenosine solid supports (Figure 3).^1^ The most practical route commences with 9-(3′-azido-3′-deoxy-*ß*-d-arabinofuranosyl)adenine **1**, which is readily accessible in two steps from commercially available 9-(arabinofuranosyl)adenine.^39^ Then, amidine protection of the adenine exocyclic NH_2_ group (**2**) and tritylation of the arabinose 5′-OH (**3**) are accomplished straightforwardly. Inversion of the configuration at C2′ (**4** and **5**) is achieved by activating the arabinose 2′-OH as the triflate, followed by substitution with potassium trifluoroacetate, which has the advantage that the resulting trifluoroacetic ester is instantaneously cleaved during workup to provide the free ribose 2′-OH. Subsequently, Staudinger–Vilarrasa^40,41^ coupling furnishes the amino acid-linked key intermediate **6**, or alternatively, this amino acid-adenosine module can be obtained in a stepwise reaction sequence by the reduction of the azido group under Staudinger conditions and isolation/purification of the amine, followed by amino acid coupling.^2,42^ Compound **6** is then transformed into pentafluorophenyl adipinic acid ester **7** and loaded onto the amino-functionalized resin to provide solid support **8**. This synthetic route has proven robust and efficient. Most of the 20 proteinogenic amino acid-charged adenosine supports have been synthesized, in addition to several supports that contain d-configured and/or nonproteinogenic amino acid residues.^2,42−51^ Of note, for the solid-phase synthesis of 3′-peptidyl-tRNA mimics that lack the 2′-OH and offer a 2′-deoxyribose instead, we introduced a suitable solid support that is attached via the exocyclic amino group of the nucleobase to resins, functionalized as *N*,^6^*N*^6^-glutaryl-9-[(2-hydroxyethoxy)methyl]) 3′-amino-2′,3′-dideoxyadenosine.^1^

Furthermore, we (R.M. and co-workers) diversified the 3′-aminoacylamino-3′-deoxyadenosine solid-phase supports to meet the challenge of *N*-methylation, *O*-phosphorylation, and phosphonation of amino acid side chains of such conjugates. In particular, to explore the biosynthesis of selenocysteine-tRNA, we introduced protocols for the synthesis of methylated, phosphorylated, and phosphonated 3′-aminoacyl-selenocysteine-tRNA mimics allowing direct methylation of the α-amino group as well as the phosphorylation directly on the *N*^α^-seryl- or *N*^α^-threonyl 3′-amino-3′-deoxyadenosine-functionalized solid support. Also, phosphono aminobutyric acid Abu(p), a common analog of *O*-phosphorylated serine, has been introduced as a functional moiety of adenosine-modified solid supports for synthesizing 3′-peptidyl-tRNA mimics.^45^

The sizes of the conjugates that have been reported for the solid-phase approach are in the range of 3 to about 30 nucleotides and 1 to 5 amino acids; typically, the number of nucleotides exceeds (or is equal to) the number of amino acids. These conjugates were purified by anion exchange chromatography.^1,2,42−51^ Aggregation was not observed, even when the peptide unit exclusively consisted of hydrophobic amino acids.

### 2.2 Formylmethionylated Peptidyl-tRNA Mimics

**Figure 4:**
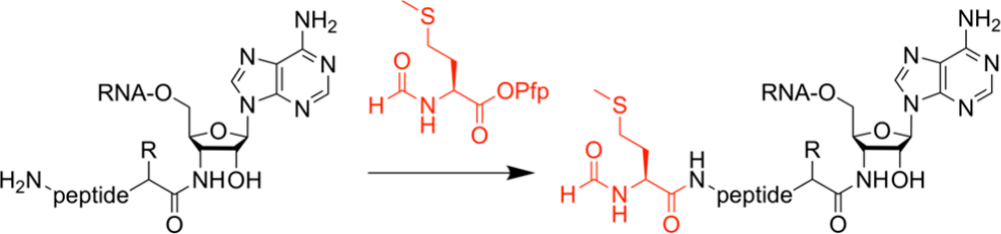
Coupling of *N*^α^-formyl methionine on deprotected peptidyl-tRNA conjugates in solution (Pfp pentafluorophenyl).^51^

Although the solid-phase approach described above allows for high flexibility of both the peptide and the RNA sequence, the protection group strategy has a limitation with respect to generating the characteristic *N*^α^-formyl methionine terminus because the formyl group of the conjugate synthesized at the solid support is cleaved during the final basic deprotection/release step. A solution to this problem has been described recently by demonstrating that the coupling of appropriately activated *N*^α^-formyl methionine (as pentafluorophenyl ester) to a completely deprotected (“free”) peptidyl-RNA conjugate is selective and highly efficient (Figure 4).^51^ A further advantage of this approach is that methionine oxidation is circumvented. Usually, during solid-phase synthesis of the RNA moiety, the oxidation of thioether to sulfoxide (see refs (1) and (43)) is at least partially observed for methionine-containing conjugates because of the repeated exposure to I_2_ solutions required for P(III)-to-P(V) oxidation to generate the RNA phosphate backbone. However, if the N-terminal methionine is coupled after solid-phase synthesis, it is not exposed to the oxidative reaction conditions. The only limitation of the *N*^α^-formyl methionine pentafluorophenyl ester approach concerns lysines that contain a primary amino group at their side chains; these become formylmethionylated without a proper protection strategy. Conceptually, lysine-containing conjugates are accessible by this approach only if an orthogonal (e.g., photolabile) protection group is applied at the *N*^ε^ position that is finally cleaved after fMet has been coupled to the N-terminus of the peptide.^52^

### 2.3 Native Chemical Ligation: Arginine-Containing Peptidyl-tRNA Mimics

A substantial challenge for the solid-phase synthesis of peptidyl-tRNA mimics concerns target sequences that contain arginine. Typically, in Fmoc chemistry, the arginine side chain is protected with the acid-labile *N*^ω^-(2,2,4,6,7-pentamethyl-dihydrobenzofuran-5-sulfonyl) (Pfb) group and, less frequently, with benzyloxycarbonyl (Z) or NO_2_ groups. All of these protections are troublesome with respect to the required detritylation step during RNA solid-phase synthesis (Pfb) or during deprotection of the peptidyl-RNA conjugate and cleavage from the solid support (Z, NO_2_). Only very few reports are found for appropriate orthogonal (e.g., photolabile, ß-eliminating) guanidine side chain protections suitable for Fmoc solid-phase peptide synthesis.^52,53^

**Figure 5:**
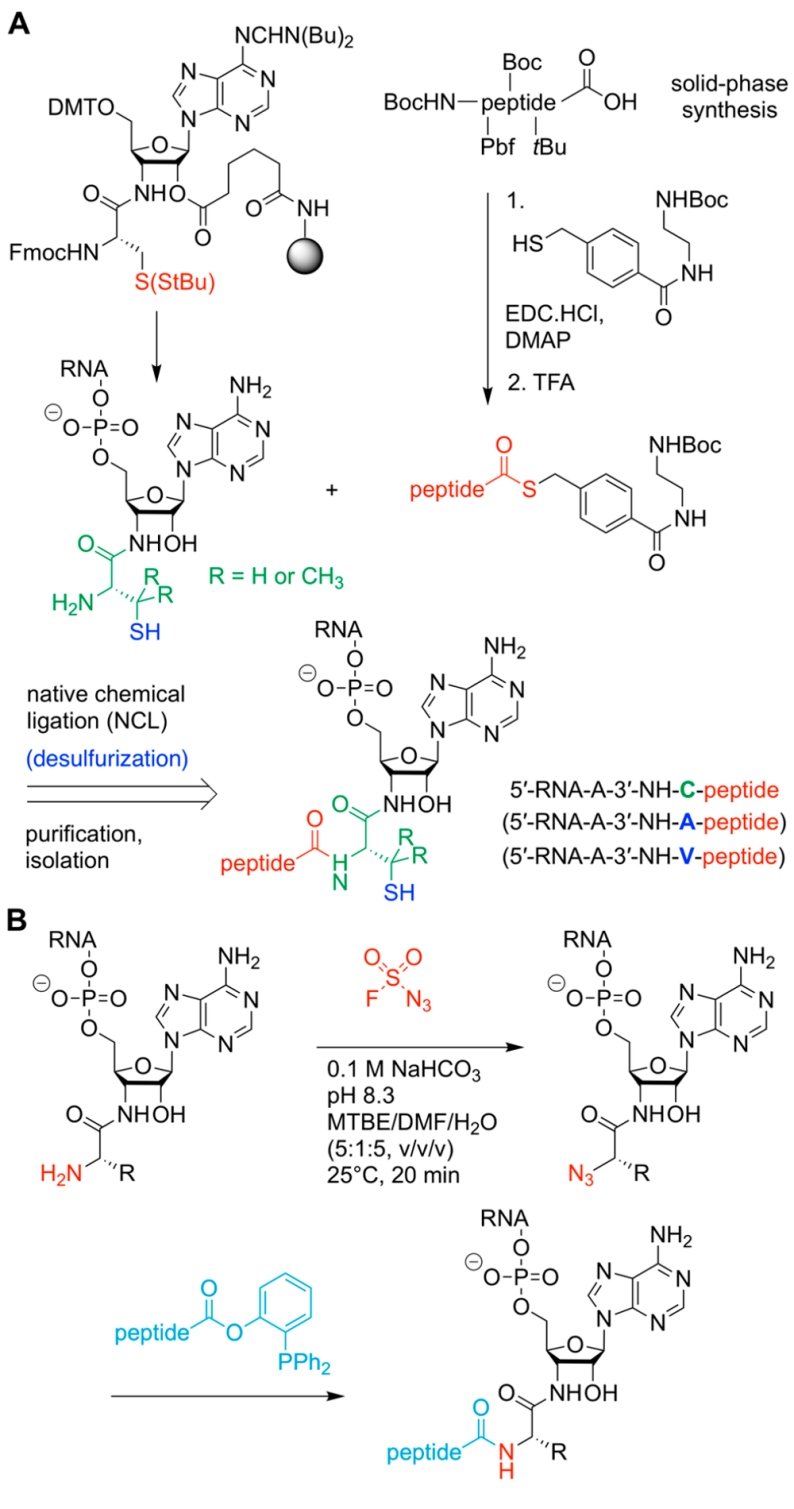
Chemical ligation of 3′-peptidyl-tRNA analogs. (**A**) Native chemical ligation of 3′-cysteinyl-RNA and peptide benzylthioesters;^2,42^ desulfurizatioin increases sequence diversity.^47^ (**B**) Amine-to-azide conversion on peptidyl-RNA by metal-free diazotransfer allows subsequent Staudinger ligation with appropriately activated amino acids or peptides.^58^

Therefore, we (R.M. and co-workers) developed a convergent route to generate arginine-containing peptidyl-tRNA mimics involving native chemical ligation (NCL) (Figure 5).^2,42^ NCL was initially designed to link unprotected peptide fragments under mild reaction conditions.^54^ The process involves a reaction between a weakly activated C-terminal thioester and an unprotected N-terminal cysteine residue.^54−57^ The thermodynamic strength of an amide bond over a thioester bond is the driving force behind this reaction, which is made possible through proximity-driven S-to-N acyl migration.

To obtain peptidyl-tRNA conjugates using NCL, one fragment is an RNA–peptide conjugate providing the 3′-terminal cysteine moiety, whereas the other fragment, the arginine-containing peptide, is functionalized as a thioester.^2^ More precisely, 4-(*N*-(2-aminoethyl)carbamoyl)benzyl thioesters (ABT) are applied because of the increased solubility in aqueous buffer solutions needed for the NCL reaction with cysteinyl-RNAs. Based on the above-described synthetic approach, the 3′-cysteinylamino-3′-deoxyadenosine-modified solid support is readily accessible (Figure 5A).^2,42^ Thereby, the thiol cysteine side chain is masked as a disulfide with the *S*-StBu group. This group is sufficiently stable during aqueous I_2_ treatment (low concentration) that is required for P(III)-to-P(V) oxidation within each RNA coupling cycle and is readily cleaved *in situ* with tris(carboxyethyl)phosphine. Subsequently, NCL is performed in good to excellent yields. To increase the diversity of the peptide sequences that the NCL approach can achieve, we (R.M. and co-workers) further developed a mild radical desulfurization protocol that allows the transformation of cysteine to alanine or, if penicillamine is used for the NCL reaction, to valine (Figure 5A).^47^ Recently, we (R.M. and co-workers) have expanded the approach to cysteine-free ligation methods. This became possible by metal-free diazo transfer on peptidyl-RNA conjugates using the diazotizing reagent fluorosulfuryl azide (FSO_2_N_3_), which quantitatively transforms the terminal primary amino group (N^α^) into an azide without affecting the nucleobase amino groups (Figure 5B).^58^ The obtained azido-peptidyl-RNA can then be further processed utilizing well-established bioorthogonal reactions, such as Staudinger ligations, to extend the peptide moiety of the 3′-peptidyl-tRNA mimics.^58,59^

### 2.4 Tailored Full-Length Peptidyl-tRNA Mimics

With the solid-phase synthesis approach for stable 3′-peptidyl-tRNA mimics described above, the achievable RNA moiety length is roughly the same as for standard RNA synthesis, meaning that 50 to 60 nt are well accessible, and even full-length RNAs of 76 nt and more could be synthesized using the protection groups that allow for mild deprotection conditions.^60,61^ Nevertheless, severe limitations are encountered for the RNA solid-phase synthesis if all natural post-transcriptional nucleoside modifications need to be present in the RNA portion of the final peptidyl-tRNA conjugate. Some of the most frequently occurring tRNA modifications^62^ and, in particular, a combination of them (e.g., 7-methyl guanosine (m^7^G), 1-methyladenosine (m^1^A), 3-methylcytidine (m^3^C), dihydrouridine (D), wybutosine (yW), queuosine (Q), lysidine (k^2^C), 2-methylthio threonyl adenosine (m^2^t^6^A), 3-(3-amino-3-carboxypropyl)uridine (acp^3^U), 5-aminomethyl-2-thiouridine (mn^5^s^2^U), etc.) are very difficult to include in the same RNA by means of chemical solid-phase approaches.^63^

**Figure 6:**
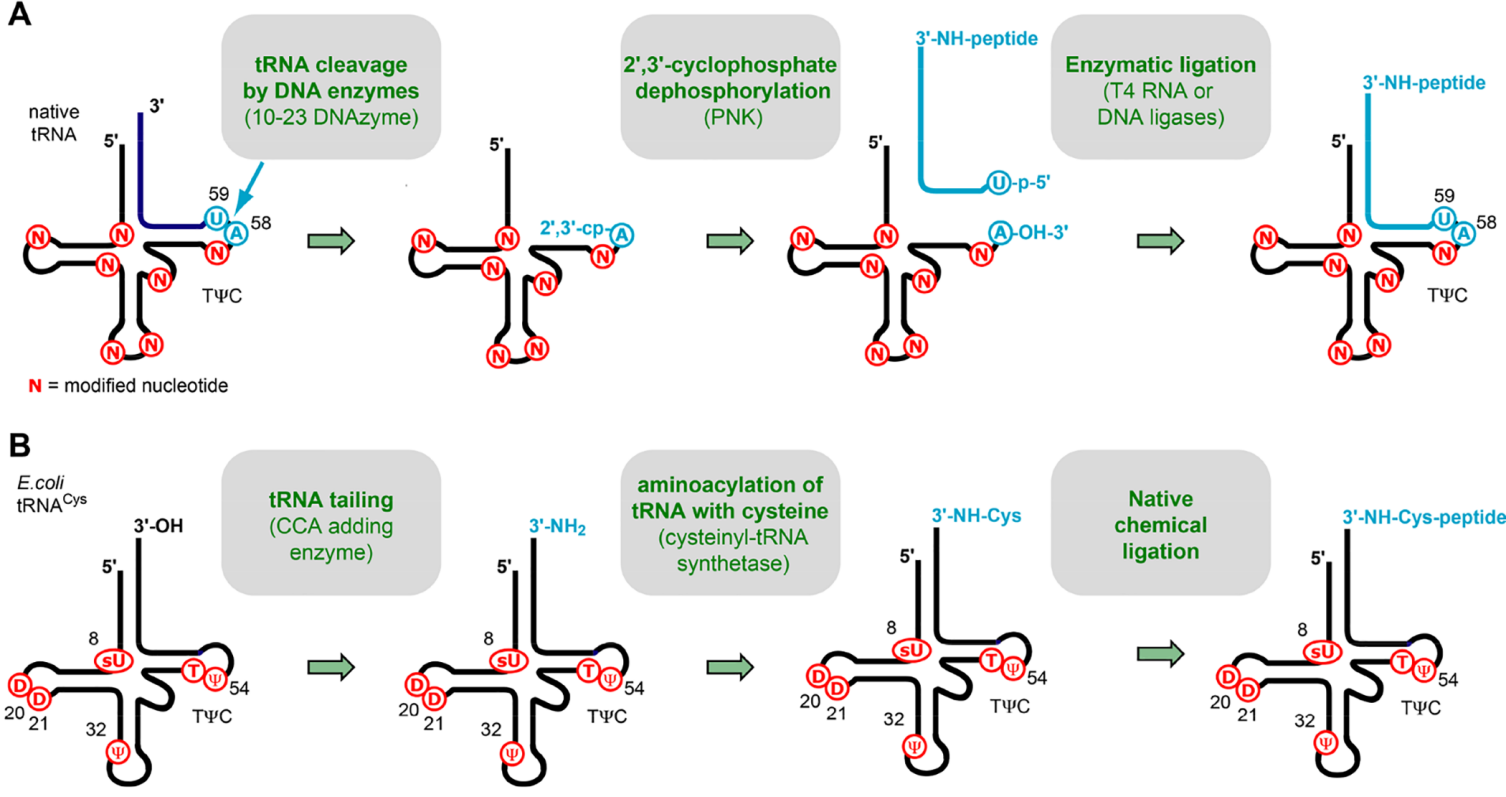
Experimental approaches to generate amide-linked full-length peptidyl-tRNAs containing all native modifications using (**A**) DNA enzymes^3^ or (**B**) native chemical ligation.^21^ Typical positions of natural post-transcriptional modifications are shown in red. The 3′-terminal sequences (18nt) of tRNAs are usually unmodified (from position 59 onward) with few exceptions (see ref (65)); the arrow (cyan) indicates the intended DNA-enzyme-mediated tRNA cleavage site and the site of ligation (to synthetic 3′-peptidylamino-RNA conjugates). Modified nucleosides, N; cyclophosphate, cp; and phosphate, p.

For the target conjugates with a hydrolysis-resistant amide linkage between the peptide moiety and a full-length tRNA containing all natural nucleoside modifications, we (R.M. and co-workers) developed a strategy (Figure 6A)^3,64^ using tRNAs from natural sources that are site-specifically cleaved within the TΨC loop by using DNA enzymes to obtain defined tRNA 5′-fragments carrying all natural modifications. After dephosphorylation of the 2′,3′-cyclophosphate moieties from these fragments, they are ligated to the respective 3′-peptidyl-tRNA termini that were prepared following the lines of the above-described solid-phase synthesis. This approach can efficiently produce nonhydrolyzable 3′-peptidyl-tRNA conjugates possessing all natural nucleoside modifications. A limitation of the approach is that only a few tRNAs from natural sources are commercially available in uniform quality. The isolation, purification, and characterization of a single tRNA species (including the determination of the actual modification levels) can be laborious and time-consuming.

## 3 Engineering of tRNA Termini to Generate Stable Peptidyl-tRNA Mimics

**Figure 7:**
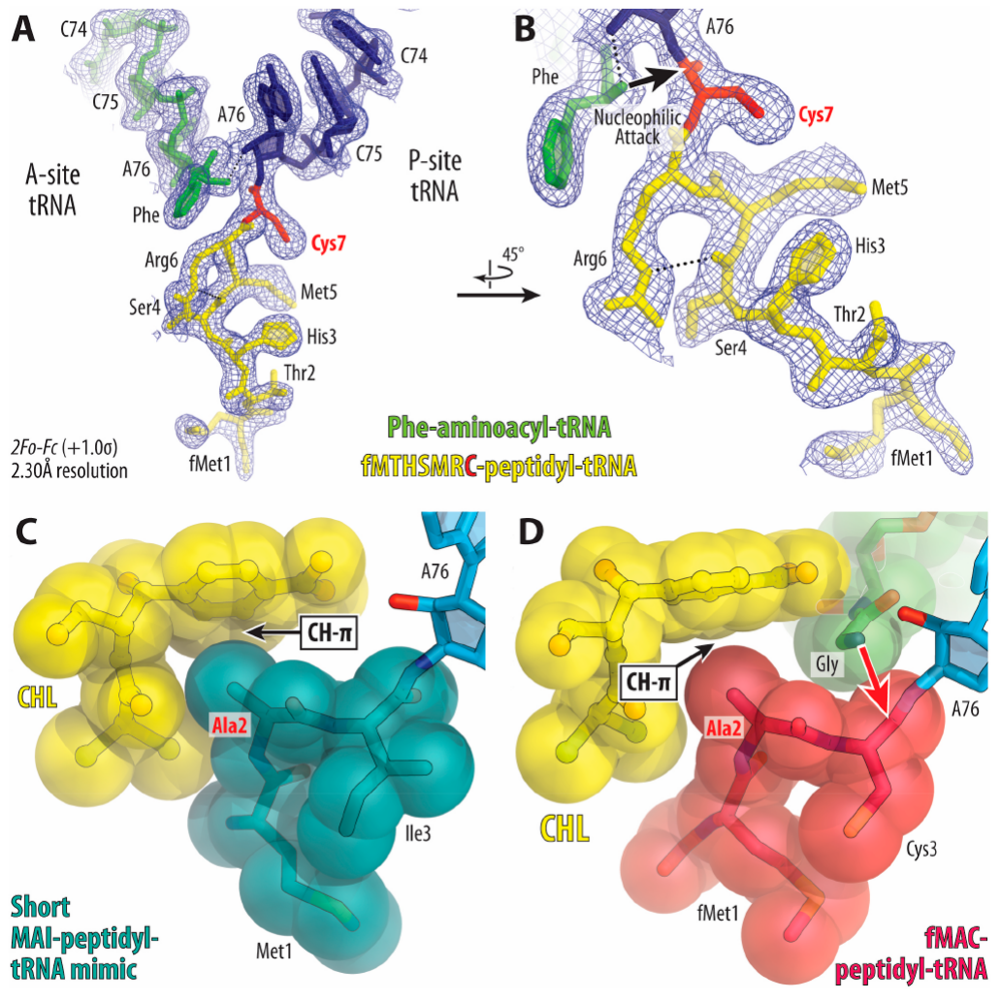
Exemplary structures of 70S ribosome carrying synthetic peptydyl-tRNAs or short mimics that were introduced in *trans*. (**A**, **B**) Close-up views of the ribosome-bound P-site fMTHSMRC-nh-tRNA_i_^Met^ (blue with peptide highlighted in yellow) and A-site Phe-nh-tRNA^Phe^ (green) and its respective electron density map (blue mesh) in the peptidyl transferase center of the 70S ribosome. The C-terminal cysteine residue of the peptide moiety is highlighted in red. (**C**, **D**) Structures of chloramphenicol (yellow) in the context of arrested (**C**) and nonarrested (**D**) ribosome nascent chain complexes reconstituted with short tripeptidyl-tRNA mimic MAI-nh-ACCA (**C**) or full-length fMAC-peptidyl-tRNA (**D**). The structures are from PDB entries: 8CVL (**A**, **B**), 7RQE (**C**), and 7U2J (**D**).

Convenient large-scale preparation of amide-linked full-length peptidyl-tRNAs without the need for chemical solid-phase synthesis of the RNA part (using the type of solid supports described above) follows a recent biochemical protocol developed by one of our groups (Y.S.P. and co-workers) (Figure 6B).^20^ The overall procedure comprises three steps: (i) tRNA-tailing to replace the 3′-terminal adenosine-3′-OH of the CCA-end with its amino-substituted adenosine-3′-NH_2_ analog;^24,66−69^ (ii) enzymatic charging of the tailed 3′-NH_2_-tRNA with cysteine by the aminoacyl-tRNA-synthetase;^66−69^ and (iii) native chemical ligation of the (commercially available) thiobenzyl-activated peptide with cysteinyl-tRNA to yield the final product.^21^ Although the usable tRNAs are restricted to initiator tRNA_i_^Met^ and elongator cysteine-specific tRNA^Cys^ and the peptide sequences must always have cysteine at the C-terminus, a wide range of structural studies that aim at exploring the interactions between the peptide chain and the ribosomal tunnel can be fueled by conjugates made this way (Figure 7A,B). Most importantly, synthetic peptidyl-tRNAs can be efficiently complexed to the ribosome *in vitro* and yet represent a functionally significant state of the PTC. Using nonhydrolyzable peptidyl-tRNAs, we (Y.S.P. and co-workers) determined the first set of structures of nonarrested (nonstalled) RNCs in the functional prepeptidyl transfer state (Figure 7A,B) at the highest resolution available to date (2.3–2.5 Å).^21^ These structures provided several new, unexpected insights into the mechanism of PTC functioning. For example, we (Y.S.P. and co-workers) revealed a previously unknown role of the ribosome in stabilizing the C-terminal portion of the growing peptide chain within the PTC through multiple H-bonds between the main chain of the nascent peptide and the universally conserved and essential PTC nucleotides. The availability of such peptidyl-tRNAs opens many directions for the structural studies of RNCs under stalling and nonstalling conditions. This chemoenzymatic approach is convenient for researchers who do not have direct access to chemical solid-phase synthesis instruments because it utilizes only commonly available equipment, affordable chemicals, and universally available commercial peptide synthesis services and therefore could be employed virtually by any biochemistry laboratory. We also note that charging of the 3′-amino-tailed tRNA with various amino acids is not limited to the use of aminoacyl-tRNA-synthetases and can also be achieved by flexizymes (flexible tRNA-aminoacylating ribozymes).^70^

## 4 Applications of 3′-Amide-Linked Peptidyl-tRNA Mimics

Conjugates synthesized by the above-described approaches find applications in structural studies focusing on understanding the mechanisms of antibiotic action. For instance, macrolide antibiotics arrest protein synthesis at the Arg/Lys-X-Arg/Lys (+X+) motifs. In a collaborative effort with the Mankin laboratory, we (R.M. and co-workers) found that the charge and size of this motif’s key amino acid side chains make peptide bond formation inefficient. Antibiotics greatly magnify the problem of these intrinsically difficult donor–acceptor pairs.^44,71^

Furthermore, using a series of tripeptidyl-tRNA mimics (Figure 7C), we (Y.S.P., R.M., and co-workers) provided a detailed view of the molecular mechanism and rationalized the context-specific action of the classic PTC-acting antibiotic chloramphenicol (CHL).^4^ Recent discoveries have shown that CHL preferentially arrests translation when the ribosome needs to synthesize particular amino acid sequences. By determining the high-resolution structures of the ribosome in complex with short peptidyl-tRNA mimics, we found that, by forming direct interactions with the ribosome-bound CHL, a nascent peptide with alanine, serine, or threonine in the penultimate (−1) position provides an additional binding interface for the CHL molecule, thereby increasing its affinity for the ribosome and inducing its action (Figure 7C). Other residues (except for glycine) in the same (−1) position of the nascent peptide sterically interfere with CHL binding. The CHL-induced ribosome stalling occurs when an amino acid residue of the incoming aa-tRNA is unable to displace the tightly bound CHL molecule from its canonical binding site, which happens when it is stabilized by interactions with the proper (−1) residue of the growing peptide, and therefore it cannot be incorporated into the ribosomal A site, making peptide bond formation unattainable. In contrast, the transpeptidation reaction is insensitive to CHL inhibition if the incoming aa-tRNA carries a glycine residue. By determining the structure of CHL in complex with the ribosome carrying A-site glycyl-tRNA and P-site fMAC-peptidyl-tRNA, we (Y.S.P. and co-workers) discovered that, due to the lack of a side chain, glycine is the only residue that can coexist in the ribosomal A site together with the CHL molecule (Figure 7D).^72^ Unlike previous studies of CHL bound to vacant ribosomes, our structures featuring peptidyl-tRNA mimics show that the drug’s binding site is formed not only by the ribosome alone but also by the growing polypeptide; therefore, the shape of the drug-binding pocket is continuously changing during translation,^4^ Thus, a new paradigm-shifting concept emerged from our studies that drug binding is controlled not only by the ribosome but also by the ribosomal substrates. Besides direct clinical implications for developing next-generation antibacterials, the significance of this new knowledge is that it is instrumental for a better, deeper, and more accurate understanding of the most fundamental functions of the ribosome.

In another study, we (Y.S.P. and co-workers) have determined the first crystal structure of the ribosome in complex with a d-aminoacyl-tRNA analog.^48^ Despite the long-standing knowledge that d-amino acids slow the rate of ribosomal peptide synthesis, it took more than 40 years for the first structure that explains the poor reactivity of d-amino acids. The structure reveals that, similarly to l-amino acids, a d-amino acid binds to a ribosome by inserting its side chain into the ribosomal A-site cleft.^48^ However, this binding mode does not allow optimal nucleophilic attack of the peptidyl-tRNA by a d-amino acid’s reactive α-amino group (because of the distinct C^α^ configuration). Altogether, our structural analysis provided insight into the ancient molecular mechanism that allows the ribosome to discriminate against the chirality of an incoming amino acid and prevent the incorporation of d-amino acids into natural proteins.^48^

Moreover, stable prolyl-tRNA mimics have played a significant role in investigating the effect of proline on peptide bond formation in the PTC.^49^ As a ribosome substrate, proline reacts markedly more slowly when compared with other amino acids both as a donor and as an acceptor of the nascent peptide. Crystal structures of the eukaryotic ribosome bound to analogs of mono- and diprolyl-tRNAs provided high-resolution snapshots of the PTC, showing that the cyclic nature of the proline residue prevents the same placement of this residue as for the other amino acids. Moreover, steric interference affects the position and conformation of the diprolyl-containing nascent peptide chain in the NPET, rationalizing the observed poor reactivity of such substrates in the PTC.^49^ These observations further revise an old dogma that amino acids bind the active site of the ribosome uniformly by showing that proline has a binding mode distinct from those of other amino acids.

Finally, stable alanyl-tRNA mimics have been successfully applied in the study on tRNA mischarging that is often corrected through the activity of specialized editing domains present in some aminoacyl-tRNA-synthetases or via single-domain trans-editing proteins.^50^ ProXp-Ala is a trans-editing enzyme that edits the product of Ala mischarging by prolyl-tRNA synthetase. Deacylation assays using substrate analogs reveal that size discrimination is the only selectivity component. In a broad systematic study, NMR spectroscopy was used to map specificity determinants.^50^ Chemical shift perturbations induced by an uncharged tRNA^Pro^ acceptor stem mimic, microhelixPro, or a nonhydrolyzable mischarged Ala-microhelixPro substrate analogue identified residues important for binding and deacylation. The data obtained revealed the structural dynamics of the system that are crucial for the recognition and selection process of the mischarged analog. Overall, this study illuminated strategies such as a trans-editing enzyme used to ensure the acceptance of only mischarged Ala-tRNA^Pro^.

## 5 Heterocyclic- and Squarate-Linked Peptidyl-tRNA Mimics

**Figure 8:**
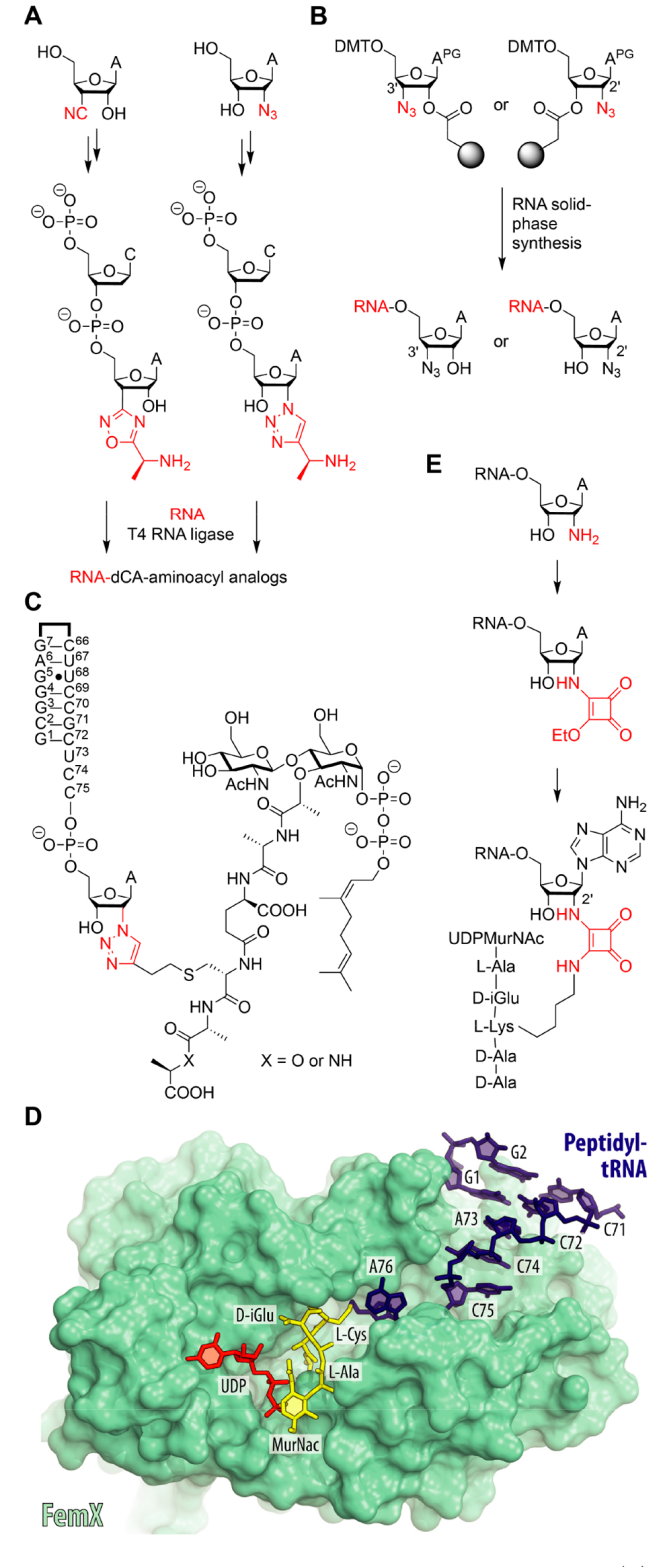
Alternative 2′ and 3′ linkages for peptidyl-tRNA mimics. (**A**) Oxadiazole- and triazole-modified dimers for enzymatic ligation to RNA. (**B**) The shown 3′-oxadiazole- and 2′-triazole-linked peptidyl-tRNA analog inhibits FemX_W_ transferase that participates in peptidoglycan synthesis and was extensively studied structurally. (**C**) Access to RNA with 2′- and 3′-azido-modified 3′ nucleoside termini is straightforward using standard phosphoramidite chemistry on the depicted solid supports. (**D**) Structure of FemX protein (teal) in complex with the 2′-fluoro-ribose (2′F-RNA) bisubstrate analog of peptidyl-tRNA (PDB entry 7Z6A). (**E**) High yielding, stepwise transformation via squarate linkage for peptidyl tRNA analogs.

Alternative to amide-linked peptidyl-tRNA conjugates, another type of hydrolysis-resistant linkage has been proven valuable in biochemical and structural studies of cellular processes that involve charged tRNAs (M.E.-Q. and co-workers). These include oxadiazole^73^ and triazole linkages,^74−77^ mimicking esters in the 3′ or 2′ position of the 3′-terminal ribose of the aminoacyl-tRNA. The oxadiazole can be introduced into the 3′-position of adenosine, and the corresponding RNA can be obtained following a chemical-enzymatic approach using classical phosphoramidite chemistry to produce the 3′-modified dinucleotides followed by enzymatic ligation using T4 RNA ligase^73^ (Figure 8A). The triazole-containing counterpart can be obtained using the same strategy^75,76^ or by a postsynthetic approach applying click chemistry on RNA with 3′-(or 2′-)azido termini,^78−80^ which themselves are easily accessible by standard RNA solid-phase synthesis^78,81−85^ (Figure 8B).

These stable analogs have been successfully used for mechanistic and structural studies of Fem transferases that play a crucial role in bacterial cell wall synthesis. These enzymes require alanyl-tRNA^Ala^ as a substrate (Figure 1E). The accessibility of the two stable regioisomers (2′- and 3′-triazole-RNA)^74−76^ enabled the analysis of the tRNA regiospecificity^75^ and revealed the ability of the enzyme to bind both regioisomers of Ala-tRNA^Ala^. This provides adequate access to activated alanine for peptidoglycan synthesis, knowing that this enzyme competes for the same pool of Ala-tRNA^Ala^ as the ribosome. More recently, these stable aa-tRNA molecules were used to investigate the participation of tRNA^Gly^ as acceptors for protein and cell-wall peptidoglycan synthesis in *Staphylococcus aureus*.^80^ Going further in the structural study of these enzymes, the amino acid part at the 2′ position was replaced by peptidyl moieties mimicking the growing peptidoglycan (Figure 8C). These (triazole)-peptidyl-RNA conjugates were synthesized by using click chemistry in a late-stage functionalization. They were tested as inhibitors of the Fem family of enzymes and showed picomolar activity for the Fem transferases of *Weissella viridescens*(86) and *S. aureus*.^79^ In the latter case, the RNA is connected to a lipid-carbohydrate-peptidyl conjugate by click chemistry.^79^ The binding of triazole-containing peptide-RNA conjugates to Fem enzymes was successfully characterized by X-ray crystallography (Figure 8D). Of note, conjugates comprising nucleic acid alternatives (xenonucleic acid), such as 1,5-anhydrohexitol nucleic acid (HNA), 2′-fluoro-arabinonucleic acid (FANA), or 2′-fluoro RNA), were well tolerated, making them attractive inhibitors for biomedical experimentation.^88^ All of these examples show the potential of stable aminoacyl-tRNA or peptidyl-tRNA to explore and deepen our understanding of nonribosomal peptide synthesis.

Another useful stable linkage between the tRNA terminus and the peptide moiety employs squarate (Figure 8E).^87^ The chemical strategy used to introduce a squarate linkage between the RNA and the peptide moiety is based on RNA solid-phase synthesis and 3′-azido/3′-amino postfunctionalization to introduce an electrophilic site at the 3′ end of RNA. The squarate diester can be used as an electrophile-enabled sequential amidation, providing asymmetric squaramides with high selectivity. The squarate-RNAs are then reacted with an amine of a peptide, in the specific case with the lysine side chain of UDP-MurNAc-pentapeptide, a peptidoglycan precursor used by the aminoacyl-transferase FemX_Wv_ for the synthesis of the bacterial cell wall (Figure 8E). The peptidyl-RNA obtained with squarate-RNA and unprotected UDP-MurNAc-pentapeptide efficiently inhibits FemX_Wv_. The squarate unit also promoted specific cross-linking of RNA to the catalytic Lys of FemX_Wv_ but not to related transferases that recognize different aminoacyl-tRNAs. The specificity of these enzymes is essential for bacteria since misincorporated amino acids can act as chain terminators and block peptidoglycan polymerization.^89^

## 6 Concluding Remarks

Significant steps forward have been made for the synthesis of 3′-peptidyl tRNA mimics, and almost any desired target sequence is now accessible with the combination of methods developed over the last 15 years and reviewed in this Account. Nevertheless, there is room for further development and improvements. The most advisible path is the solid-phase synthesis of peptidyl-tRNA conjugates based on the solid support of type **8** that integrates the C-terminal amino acid of a target peptide. However, obviously, this requires individual support for each of the 20 essential amino acids. Therefore, from the perspectives of economics and synthetic flexibility, it would be desirable to have a universal solid support that allows coupling of the C-terminal amino acid directly to the amino group of 3′-amino-3′-deoxyadenosine. The difficulty of such an approach, however, is that the typically applied acyl tether from the 2′-O of 3′-amino-3′-deoxyadenosine to the solid support will not work reliably because of migration of the acyl group to the (deprotected) free 3′-amino functionality that is to be charged with the first amino acid in the subsequent step. To solve this problem, we currently evaluate concepts that focus on 3′-amino-3′-deoxyadenosine supports containing a photocleavable linkage between the ribose 2′-O and the solid support, which, in principle, should allow coupling of any amino acid to the deprotected 3′-amino 3′-deoxyribose functionality.

The example with CHL tells us that structural models showing how various antibiotics interact with vacant bacterial ribosomes can provide incomplete or misleading information because the key interactions of a drug and the ribosome might be critically dependent on the presence of ribosomal ligands, such as tRNAs. Therefore, using the functionally relevant ribosome complexes containing short- or full-length mimics of aminoacyl- and peptidyl-tRNAs makes it possible to produce principally new data highly relevant to the actual mechanism of antibiotic action. This knowledge may help the future development of antibiotics that inhibit growth and actively kill pathogenic bacteria more potently than currently available drugs. Understanding the broad bases of translation regulation by ligands bound in the nascent peptide exit tunnel may provide new ways for modulating protein synthesis in bacterial and eukaryotic cells, thereby opening new venues for developing ribosome-targeting drugs useful for a variety of human diseases.

In addition, data on the catalytic mechanism and active site structure of enzymes involved in nonribosomal peptide synthesis, such as the bacterial FemX family, should provide crucial information for the rational design of drugs that act on original targets.
